# *QuickStats:* Trends in Secondhand Smoke Exposure* Among Nonsmoking Adults, by Race^†^ and Hispanic Origin — National Health and Nutrition Examination Survey, United States, 2009–2018

**DOI:** 10.15585/mmwr.mm7006a6

**Published:** 2021-02-12

**Figure:**
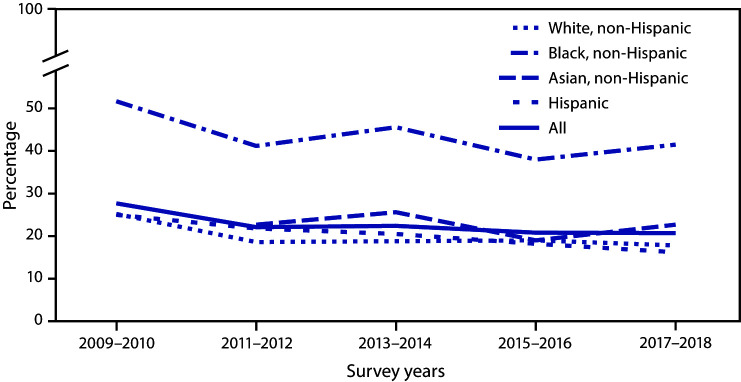
The percentage of nonsmoking adults exposed to secondhand smoke (SHS) declined from 27.7% in 2009–2010 to 20.7% in 2017–2018. During this period, decreasing trends in the percentage of persons with SHS exposure also were observed for nonsmoking non-Hispanic White, non-Hispanic Black, and Hispanic adults. There was no significant decline in the percentage of persons with exposure for nonsmoking non-Hispanic Asian adults from 2011–2012 to 2017–2018. The percentage of persons with SHS exposure was consistently higher for nonsmoking non-Hispanic Black adults throughout the period. During 2017–2018, 41.5% of nonsmoking non-Hispanic Black adults were exposed to SHS compared with 22.7% non-Hispanic Asian, 17.8% non-Hispanic White, and 16.2% nonsmoking Hispanic adults.

**Source:** Brody DJ, Faust E, Tsai, J. Secondhand smoke exposure among nonsmoking adults: United States, 2015–2018. NCHS data brief, no. 396. Hyattsville, MD: US Department of Health and Human Services, CDC, National Center for Health Statistics; 2021.

